# The Spread of the Mosquito‐Transmitted West Nile Virus in North America and Europe

**DOI:** 10.1111/1751-7915.70120

**Published:** 2025-03-04

**Authors:** Harald Brüssow, Jordi Figuerola

**Affiliations:** ^1^ Department of Biosystems, Laboratory of Gene Technology KU Leuven Leuven Belgium; ^2^ Department of Global Change and Conservation Estación Biológica de Doñana‐CSIC Sevilla Spain; ^3^ CIBER Epidemiología y Salud Publica Madrid Spain

**Keywords:** American robin, climate change, Culex mosquitoes, Flavivirus, horse epizootics, human epidemics, viral reservoir species, West Nile Virus

## Abstract

West Nile virus (WNV) disease, a mosquito‐transmitted Flavivirus infection, represents a substantial public health research interest. This virus was unknown in the Western hemisphere until it was introduced in 1999 into an immunologically naïve population. WNV caused an epizootic and epidemic in New York City. The infection then swept over North America, causing mass mortality in birds and cumulatively 60,000 human cases, half of whom were hospitalised, mostly with neurological symptoms. The virus closely resembled a goose virus isolated in Israel in 1998. Mosquitoes of the genus *Culex* were identified as the insect viral vectors. WNV can infect more than 300 bird species, but in the US, the American robin *(Turdus migratorius* ) represented the ecologically most important bird viral reservoir. Mosquito‐to‐mosquito viral transmission might amplify the viral spread, and iatrogenic WNV transmission was also observed, leading to the screening of blood products. Compared with African WNV isolates, the New York WNV isolate NY99 showed a mutation in the nonstructural protein NS3 that increased its virulence in birds and was also observed in WNV outbreaks from Romania in 1996 and from Russia in 1999. During its spread across the US, NY99 acquired a mutation in the envelope gene E that favoured viral infection in the insect vector. Europe reported 1200 annual WNV cases in 2024, with a focus in Mediterranean countries, but a northward spread of the infection to Germany and The Netherlands was also noted. Global warming is likely to affect the geographical distribution of vector‐borne infections such that people living in temperate climate areas might be increasingly exposed to these infections. Therefore, research on temperature effects on WNV transmission by *Culex* mosquitoes has become a recent focus of research. Pertinent climate aspects of WNV infections are retraced in the present review.

## Graphical abstract

The West Nile flavivirus is not only maintained in the USA in a primary infection cycle between the American robin and Culex mosquitoes but also causes incidental infections in horses and humans, causing epizootics and epidemics. The ecological conditions influencing virus transmission in North America and Europe are reviewed.

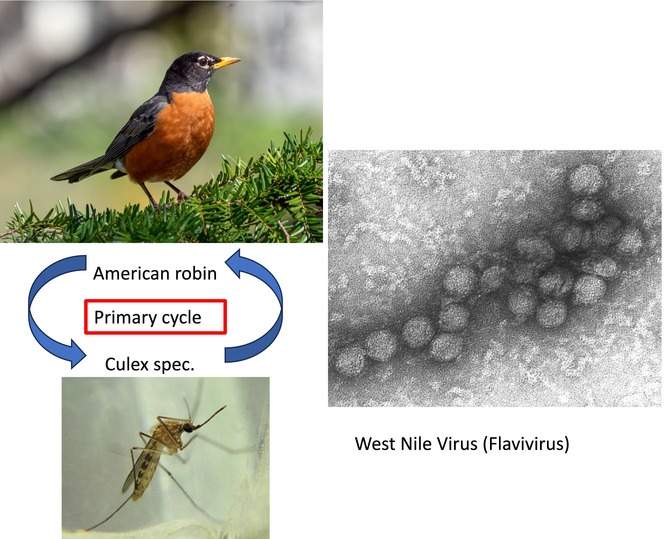

West Nile Virus (WNV), an arthropod‐borne (arbovirus) Flavivirus, is not a candidate virus for a pandemic. However, there are good reasons for interest in WNV. First, it causes neurological disease in humans associated with substantial mortality. Second, the natural history of WNV allows us to study the introduction of a ‘new’ virus into an immunologically naïve population, as occurred in North America. Third, as arboviruses cycle between replication in insect and vertebrate hosts, viral evolution is constrained by the need for replication in two very different host systems, representing challenging genetic trade‐offs. Fourth, WNV is an important veterinary pathogen of horses, and fifth, WNV infections are an intensively studied test case for the impact of climate change on the geographical spread of viral pathogens.

Within the insect‐transmitted Flaviviruses, two large groups are distinguished by phylogenetic tree analysis. One branch is tick‐borne flaviviruses; another branch is evolutionarily related, mosquito‐borne flaviviruses (Pearson et al. 2021). The latter can further be differentiated according to the mosquito vector. The *Aedes* mosquitoes are associated with flaviviruses such as Yellow Fever Virus, Dengue Virus or Zika Virus, while the *Culex* mosquitoes are associated with the flaviviruses Japanese Encephalitis Virus and WNV. The name of the latter refers to its isolation in the West Nile district in Uganda in 1937. WNV cycles in nature between *Culex* mosquitoes and birds (primary transmission cycle). Strikingly, more than 300 avian species are susceptible to WNV. WNV also infects humans, horses and other mammals as incidental, dead‐end hosts. In the past, WNV had caused sporadic human outbreaks of a mild febrile illness in Africa, Europe, the Middle East, Asia and Australia. In the 1990s, the epidemiology of WNV infections changed markedly in Europe and severe clinical courses were reported in humans. Notably, during the 21^st^ century, the virus has become endemic in Europe, and although several introductions from Africa have occurred, the virus is overwintering in its reservoirs in southern European countries, producing recurrent outbreaks (Aguilera‐Sepúlveda et al. 2024). In view of the current discussion on emerging viral diseases and One Health issues, it is instructive to retrace the recent spread of WNV infections in North America and Europe.

## 1 The Arrival of the West Nile Virus in North America

### 1.1 The 1999 New York City Outbreak

#### 1.1.1 Symptoms and Incidence Rates

Between August and September 1999, 59 patients with laboratory‐proven WNV‐associated encephalitis were hospitalised in New York City (NYC). The diagnosis was based on the detection of WNV‐specific IgM and neutralising serum antibodies as well as WNV genome detection in cerebrospinal fluid samples. In patients who died from the infection, WNV antigen was detected in the brain by immunochemistry. The NYC WNV outbreak was unexpected because this virus was neither known to circulate in the Western Hemisphere nor to cause severe neurological symptoms. The dominant clinical presentation was encephalitis in 60% of the patients (with a 30% case fatality rate), meningitis in 30% of the patients and profound muscle weakness. Risk factors for severe disease were age above 75 years (Nash et al. 2001). Historical data from Israel estimated that overt disease occurred in 1 of every 100 WNV infections but did not include severe neurological disease (Goldblum et al. 1956).

The infection rate in NYC was greater than indicated by the number of patients hospitalised with the WNV disease. Cluster serum sampling from 700 subjects living in the Queens epicentre of the NYC outbreak revealed a WNV seroprevalence of 2.6%. Seropositivity was associated with the reporting of fatigue, headache and myalgia. The seroprevalence data suggest that for the 1999 NYC outbreak, there were 8000 WNV infections, 1700 of which were associated with a febrile illness and fewer than 1% developed a severe neurological disease (Mostashari et al. 2001).

#### 1.1.2 Searching the WNV Origin

*Culex* mosquitoes and birds are the usual suspects for WNV transmission. Concomitant with the 1999 human WNV outbreak, viral encephalitis was reported in crows from NYC and several bird species from the Bronx zoo. Necropsy‐recovered material yielded, upon inoculation of embryonated chicken eggs, an infectious virus. An 11,029 nucleotide‐long RNA genome was sequenced and revealed a virus belonging to WNV lineage 1 found in Middle Eastern, Eastern European and Australian WNV isolates. The closest relative to the NYC crow WNV isolate was a virus isolated from a lethal brain infection of a goose in Israel in 1998. Over a sequenced 1.2‐kb genome segment, the NYC crow virus differed by only two nucleotides from the Israeli goose isolate (Lanciotti et al. 1999). However, there is no migratory bird flyway connecting the Eastern Mediterranean area with NYC. The authors speculated that an Eastern Mediterranean‐like WNV could have been imported to NYC with an infected illegal bird transport or by infected mosquitoes travelling on airplanes. Another research group isolated WNV from *Culex pipiens* and *Aedes vexans* mosquitoes, an American crow and a hawk sampled in September/October 1999 in Connecticut. All four isolates were nearly sequence identical and matched the crow WN‐NY99 isolate (Anderson et al. 1999). WNV cannot develop high viremia in human blood; therefore, mosquitoes cannot get infected by feeding on infected humans. Consequently, the NYC outbreak could not be introduced by an infected human traveller. Its origin could only derive from the importation of an infected avian host or a competent vector.

### 1.2 Identification of the Mosquito Viral Reservoir

**FIGURE 1:**
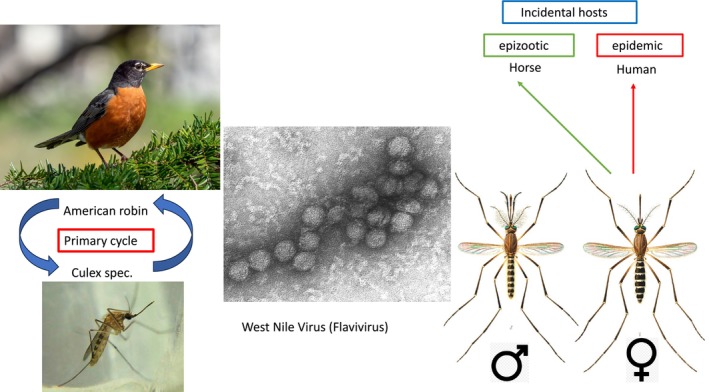
Primary infection cycle and incidental, dead‐end infections with the West Nile flavivirus. At the left side is depicted the primary infection cycle between the major US bird viral reservoir, the American robin ( *Turdus migratorius* ) and the major insect viral vector, the mosquito *Culex* spec. In the centre is an electron microscopic picture of WNV. At the right side, a male and female *Culex* mosquito pair is shown; the female also transmits the WNV to two dead‐end hosts, causing important epizootics in horses and epidemics in humans. Figure credit: Centers for Disease Control and Prevention (EM and Culex photo) http://phil.cdc.gov/phil_images/10302002/8/PHIL_2290_lores.jpg in the public domain; American robin: Rhododendrites file from Wikimedia Commons. CC BY‐SA 4.0; *Cx. quinquefasciatus* pair from E. A. Goeldi (1905) Os Mosquitos no Pará. Memorias do Museu Goeldi. Pará, Brazil. Wikipedia, in the public domain.

With respect to control measures, it makes a difference whether *Culex pipiens* or *Aedes vexans* transmit WNV. *Culex pipiens* lays eggs in wet containers (e.g., discarded tires, live plant commerce and in street drains), while *A. vexans* lays eggs in wetlands and floodwaters. To settle the question of the viral insect reservoir, 32,000 mosquitoes representing 25 species were collected in September 1999 from New York and New Jersey. The mosquitoes were sorted in 1800 pools containing either *Culex* or *Aedes* species. WNV was isolated from 15 pools, all of which were represented by *Culex* species, and six positive pools contained exclusively *C. pipiens* . No *Aedes* pool yielded WNV (Nasci et al. 2001). Other researchers collected information on the abundance, infection prevalence, vector competence and biting behaviour of 10 insect species suspected as WNV vectors. The *Cx. pipiens* species complex accounted for > 80% of the total infection risk based on its high WNV infection prevalence, the abundance of this species and the high proportion of blood meals on birds, which more than compensates for the smaller fraction of mammalian blood meals (19% for *Culex* vs. 86% for *Aedes*). According to this compound index, *Aedes* accounted for < 5% of human WNV infections in New York State (Kilpatrick et al. 2005). Further research demonstrated that the feeding pattern of *Cx. pipiens* showed seasonal changes: In early summer (May to June), they feed on the American robin ( *Turdus migratorius* ), their preferred host (Figure 1). Due to the decrease in the American robin population from its migration to the south after the breeding period, the risk of *Cx. pipiens* biting humans increases in July, which coincides with the timing of the human WNV epidemic in August to September when accounting for the incubation period between virus infections and the start of illness symptoms (Kilpatrick et al. 2006b).

### 1.3 Wild Bird Decline in the US

Tens of thousands of dead birds were reported after the spread of WNV in the US. However, distinguishing the impact of a newly introduced viral disease from the impact of environmental changes is not easy. Therefore, the evaluation of a 26‐year breeding bird survey bracketing the WNV introduction event allowed an assessment of the WNV impact on bird populations in the US against an environmental trend. Nationwide, a marked population decline was noted after 1999 for the bird species that showed before WNV introduction either population increases (American crow, chickadee, eastern bluebird) or stable populations (house wren). American crows declined by 45%, and the decline was correlated in time with the human WNV epidemic in the US. In addition, regional differences were observed in American crow populations, and their decline correlated with the number of human WNV cases. A decline of the American robin, a suspect species for initial WNV amplification, was noted in northeastern US (LaDeau et al. 2007).

When using a dataset for birds captured over two decades in North America, US researchers observed that half of the bird species were negatively affected by WNV. They distinguished two distinct patterns: species that were negatively affected by the arrival of WNV but subsequently recovered (e.g., field sparrow) and species that suffered a persistent population decline (e.g., purple finch). Compared to the human WNV epidemic, the losses from bird WNV epizootics were enormous. Over 30 million red‐eyed vireos may have died from WNV arrival, representing a 29% population decline. The study did not detect a correlation with land use pattern or phylogenetic relatedness of the most affected bird species (George et al. 2015). Lasting negative population effects of WNV epizootics were seen in only a few bird species. Lesser impact of WNV was attributed to differences in the spatial WNV transmission intensity, which allowed species with a broad range of habitats to find refuges from infection pressure (Kilpatrick and Wheeler 2019).

### 1.4 Identification of the Bird Viral Reservoir Species

To identify epidemiologically relevant bird WNV reservoirs, US researchers exposed 25 bird species to infectious mosquito bites. Marked differences were found with respect to titre and duration of viremia in the infected birds. Passeriformes birds reached serum titres of up to 10^11^ plaque‐forming units (pfu) WNV/mL serum, while Galliformes birds remained below 10^3^ pfu/mL. To qualify for competence as a reservoir species, birds must sustain a viremia greater than 10^5^ pfu/mL to infect *Cx. pipiens*. Birds with the highest reservoir competence index were blue jay, common grackle, house finch, American crow and house sparrow. Most infected birds, particularly passeriform birds, shed WNV in the faeces; some also in oral exudates. Almost all organs of infected birds contained WNV. Notably, 15 bird species could also be orally infected (owls and crows feeding on infected vertebrate prey). Infected Corvidae could infect naïve cage mates (Komar et al. 2003).

From bird decline and experimental infection data alone, it is not possible to determine the bird reservoir species for WNV transmission. One must complement these observations with the feeding preference of mosquitoes. Such data were collected from a 1‐km^2^ urban and residential area in Washington DC. The abundance of birds was counted; birds were trapped and bled to determine their WNV antibody levels. Then, mosquitoes were collected by light traps and tested for WNV RNA. Finally, the host species of the mosquito blood meal was determined by PCR sequencing of the vertebrate cytochrome b gene. It turned out that mosquitoes showed a strong feeding preference for the American robin in comparison to other species such as the house sparrow. In most sampling sites, American robins were up to 70% the preferred host for blood meals, while robins represented < 5% of the bird counts. The observation was confirmed by a 40% seroconversion rate to WNV in American robins. Numerically, a single robin infected 24 mosquitoes. American robins represent, thus, what has been dubbed superspreaders in human epidemics. The cytochrome b data demonstrated that about 10% of the blood meals were from mammals, predominantly humans. Since more than 90% of the mosquitoes were molecularly determined as *Cx. pipiens*, the infection chain *Cx. pipiens → American robin → Cx. pipiens → human* is the plausible scenario in the eastern US. While American robins are epidemic facilitators, poor amplifier bird species such as doves and starlings can dampen an epidemic as a dilution host. Their removal would therefore accelerate a WNV epidemic (Kilpatrick et al. 2006a). Data from Connecticut concurred with these observations: 40% of the *Cx. pipiens* blood meals were from the American robin, < 1% were derived from the American crow, while 6% came from mammals. Blood meals from American robins decreased from 60% in early summer to negligible levels in September/October when most individuals had migrated to the south (Molaei et al. 2006).

### 1.5 Mosquito‐To‐Mosquito WNV Transmissions

In the standard model, a susceptible mosquito bites an infected vertebrate host. The virus then multiplies within the insect vector until it infects the salivary glands, from which it is expectorated into the skin of another susceptible host during subsequent blood feeding, resulting in virus transmission. Alternative models are vertical transmission where WNV can be maintained within mosquito populations by direct transmission from an infected female mosquito to its offspring. Furthermore, after an infected blood meal, mosquitoes excrete high amounts of WNV with low infectious stability into the faeces. Mosquito pupae can become infected when experimentally exposed to infectious mosquito excreta, which might allow maintaining a WNV reservoir in mosquitoes without cycling into vertebrate hosts (Hamel et al. 2024). Other researchers have stressed the importance of nonviremic transmission of WNV. Infected *Cx. pipiens* fed simultaneously with uninfected mosquitoes on uninfected mice could transfer WNV infection to 2%–5% of naïve mosquitoes before any viremia was observed in mice. They calculated that a single infected mosquito could infect between 2 and 40 naïve mosquitoes that fed adjacent to the infected mosquito, allowing a substantial amplification of WNV prevalence in *Cx*. *pipiens* populations (Higgs et al. 2005). The ecological importance of WNV mosquito‐to‐mosquito transmissions for bird epizootics and human WNV epidemics in the US is unclear. Arthropod‐borne viruses (arboviruses) such as WNV normally perpetuate through alternating replication in vertebrate and invertebrate hosts. Arboviruses must thus maintain adequate replicative fitness in two disparate hosts in exchange for superior fitness in one host (trade‐off hypothesis). Serial passage only in the invertebrate or only in the vertebrate host or alternative vertebrate–invertebrate passages might change WNV replication fitness. In a serial passage experiment in chickens or mosquitoes, researchers observed an increased replication fitness of chicken‐passaged WNV for chickens and *Cx. pipiens* compared to the unevolved inoculum. For mosquito‐passaged WNV, replication fitness dropped dramatically in chickens and increased slightly in mosquitoes. From this outcome, the authors deduced strong and weak purifying selection in chicken and mosquito hosts for WNV, respectively (Deardorff et al. 2011).

### 1.6 Iatrogenic WNV Transmission

Mammals do not contribute to WNV infection chains: they are considered as dead‐end hosts. The reason is simple: WNV titres achieved by viremia in mammals are below the minimal infectious dose needed for a mosquito to get infected (a blood meal represents only a few microliters). However, this does not exclude the possibility that blood from infected humans does not play a role in WNV transmission. By 2002, the Centers for Disease Control and Prevention (CDC) had counted 4200 WNV infections in the US. CDC explored the risk of WNV transmission with blood donations: it searched for people who donated blood 1 week before developing WNV‐like symptoms and for people who developed fever, meningitis or encephalitis after receiving a transfusion. CDC identified 23 likely transfusion‐transmitted WNV infections. Half of them were in immunocompromised subjects. Red cells, platelets and fresh‐frozen plasma were implicated. Viremia developed in the recipients 2 weeks after transfusion. Thirteen blood recipients developed meningoencephalitis; seven died. Nine of the 14 associated blood donors reported symptoms compatible with a viral infection (fever, rash, painful eyes) around the time of blood donation. WNV titres were < 80 pfu/mL in the donors, who were all IgM negative for WNV (Pealer et al. 2003). Alerted by these observations, donor blood testing was initiated in 2003 in the US. When nearly 700,000 blood samples were screened in sets of 16 pooled samples, 183 (i.e., 1 out of 3700) samples were WNV positive by PCR tests. Further positive samples were detected when individual tests were investigated, probably indicating samples with low viral RNA titres. The mini pools detected 66% of the infected donors. Case investigations identified 17 recipients that were infected with blood samples that were negative by mini pool testing, indicating that iatrogenic infections can occur even with very low viral titres. Three quarters of the WNV RNA positive blood samples also showed antibodies to WNV. The positive blood samples were detected during the summer WNV epidemic season. During the 2004 WNV season, 1 million donations were tested, and only half as many viremic blood donors were detected compared to 2003 (Busch et al. 2005). A study from the American Red Cross concurred with these observations: In 2003, 1 in 6700 blood samples was WNV RNA positive compared with 1 in 23,000 in 2004. Positive samples clustered in the summer months, and geographically, the peak of positive samples shifted westward across the US in parallel with the westward shift of observed clinical cases. IgM antibody‐positive samples showed 60‐fold lower WNV RNA titres than IgM‐negative blood samples (Stramer et al. 2005).

WNV infection can also be transmitted by organ transplantation. An accident victim received a blood transfusion from 63 donors; one showed a low level of WNV viremia. The accident victim died, and her organs were transplanted to four organ recipients. At the time of transplantation, the organs of the donor showed a low WNV titre (< 20 pfu/mL). Nevertheless, three organ recipients developed WNV encephalitis; one recipient died with WNV antigen detected in the brain (Iwamoto et al. 2003).

Twenty percent of patients who had chronic symptoms years after hospitalisation with WNV encephalitis excreted WNV RNA but no infectious virus into the urine (Murray et al. 2010). However, other clinicians reported that urine excretion of WNV is infrequent (Baty et al. 2012).

### 1.7 Spread of WNV in North America

The ArboNET scientists merged data from avian mortality surveillance with those of human, horse and vector surveillance and observed temporally correlated WNV infections in mosquitoes, birds and humans at regional resolution (Marfin et al. 2001). CDC followed the yearly development of the WNV epidemic in North America after the 1999 NYC outbreak. In 2000, a few additional WNV cases were reported in the northeast US. In 2001, the infection had spread along the east coast, reaching Florida, but case numbers still remained low. In 2002, case numbers increased substantially and the outbreak reached Texas in the south, as well as Quebec and Ontario/Canada in the north. In 2003, the WNV infection had geographically spread further and case numbers peaked. In 2004, the outbreak reached the West coast, but case numbers decreased and showed fluctuations over the following years. Another peak was seen in 2012 with an infection focus in Texas (Murray et al. 2013). Up to 2023, the cumulative WNV case numbers in the US were 60,000, half of whom were hospitalised, mostly with neuroinvasive disease. Neurological cases showed a case fatality rate of 10%. Over the last 20 years, the highest incidence of more than 1 case per 100,000 population was observed in the Great Plains of the USA. No marked difference was seen between the sexes, but subjects younger than 40 years were less affected. Most cases were reported between July and September, with a marked August peak (Current Year Data (2024) | West Nile Virus | CDC). Until 2010, over 3 million WNV infections were estimated for the US based on seroprevalence studies, suggesting that 140–350 WNV infections resulted in one neuroinvasive disease. The highest cumulative infection incidence was observed in the Central Plains; the top state was South Dakota with 13% of the population experiencing a WNV infection, followed by Wyoming and North Dakota. At the lower end of infections, 0.1% of the population were infected in Washington state (Petersen et al. 2013). The high rates in the Great Plains might be linked with large areas of irrigated farmland, which provide breeding grounds for *Culex* mosquitoes, particularly *Cx. tarsalis*. The Central Plains experienced, in 2002, a marked WNV epizootic in horses with more than 15,000 cases, and North Dakota reported a case fatality rate of 22% in affected horses (Ndiva Mongoh et al. 2008).

Surprisingly, despite a few WNV cases of introduced infections in South America (Morales et al. 2006), no infection waves were reported in birds, horses or humans, although these countries provide suitable ecosystems (Komar and Clark 2006), except that the geographical distribution *of Cx. tarsalis* does not extend to South America (Shocket et al. 2020).

### 1.8 Phylogenomics

**FIGURE 2:**
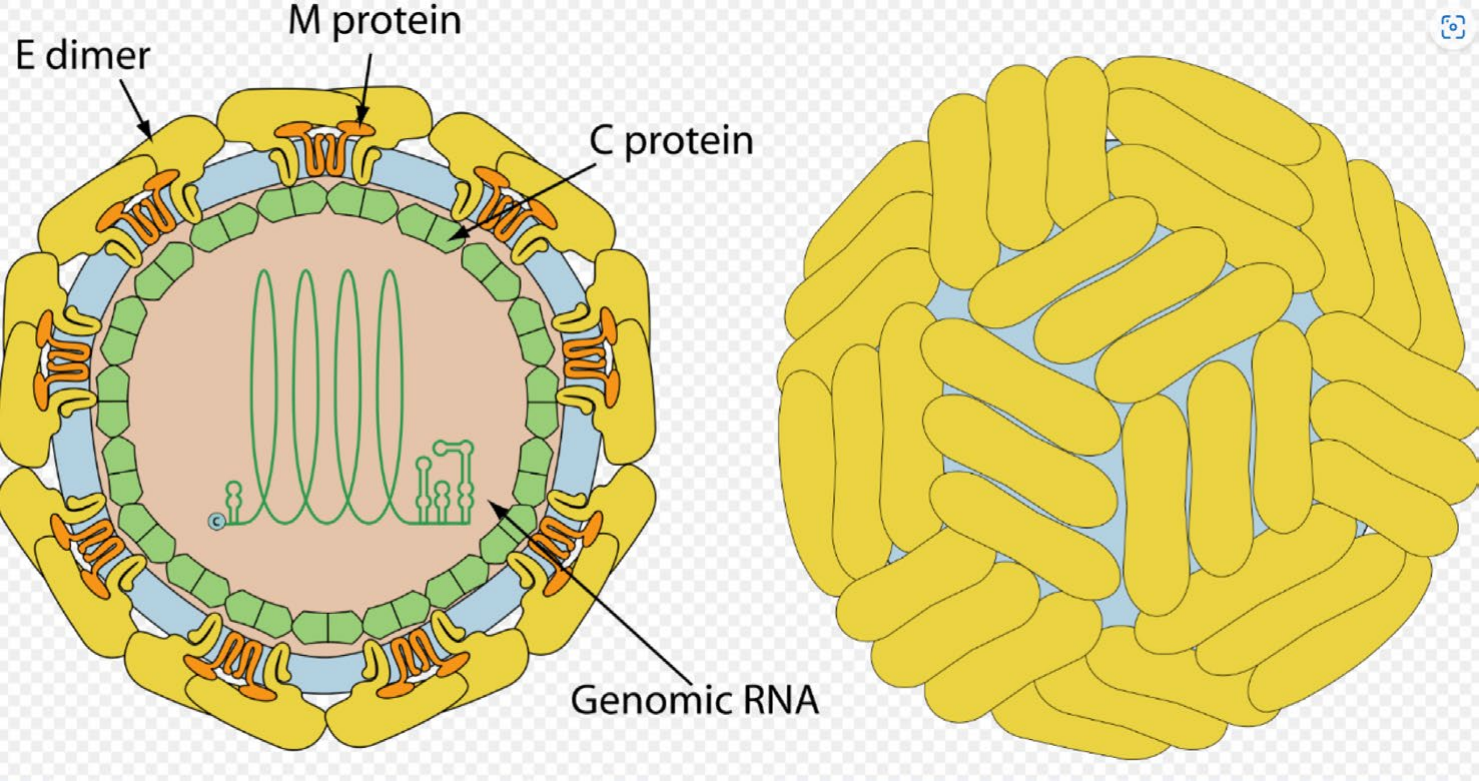
Structure of a typical Flavivirus (here a Zikavirus); the left shows a cut view of the virus with major structural proteins and viral RNA in the core (light blue lipid membrane derived from the infected cell); the right shows the surface view. Figure credit: ViralZone, SIB Swiss Institute of Bioinformatics, see https://viralzone.expasy.org/ CC BY 4.0.

Tools were developed to follow pathogen spread and evolution using genomic epidemiology approaches (Nextstrain). This tool was also applied to WNV infections in North America (Davis et al. 2005; Hadfield et al. 2019; Ronca et al. 2021). WNV genomics data from mosquitoes and birds showed that the virus from the NYC 1999 outbreak travelled with a speed of 1000 km per year from the East to the South, reaching Florida in 2001, and then to the West, reaching Washington state in 2002 and California in 2003. During travelling, WNV accumulated genetic diversity displaying a ‘bush‐like’ tree topology, meeting only a few geographical barriers. Spread was apparently favoured by migratory birds and a large trucking industry. The requirement to maintain genetic fitness for efficient replication in two very different animal hosts imposes constraints on the virus evolution such that WNV shows lower rates of genome diversification compared to RNA viruses in general. The NYC outbreak strain NY99 still reached the Mississippi river unaltered. In 2001, a mutant WN02 emerged with a single aa replacement in the envelope protein E (E‐V159A) that became dominant in 2003 and marked the westward spread of WNV (Figure 2). The mutation could be adaptive since a higher proportion of *Cx. pipiens* mosquitoes became infected and transmitted WNV after feeding on WN02 than on NY99 (Moudy et al. 2007). In addition, peak viremia titres in experimentally infected house sparrows were 10‐fold higher with WN02 than with NY99, suggesting a selective advantage for the variant virus also in birds. The researchers also noted that the peak viremia titre for NY99 decreased in sparrows between 2000 and 2015, in parallel with increasing bird survival rates, indicating resistance development in the avian host and suggesting adaptive evolution in both WNV and sparrows (Duggal et al. 2014). Later, another mutant derived from WN02 emerged, called SW03, showing two additional mutations in nonstructural proteins NS4A and NS5 (NS4A‐A85T and NS5‐K314R). In the US, both WNV strains coexisted and even co‐circulated in the same region.

**FIGURE 3:**
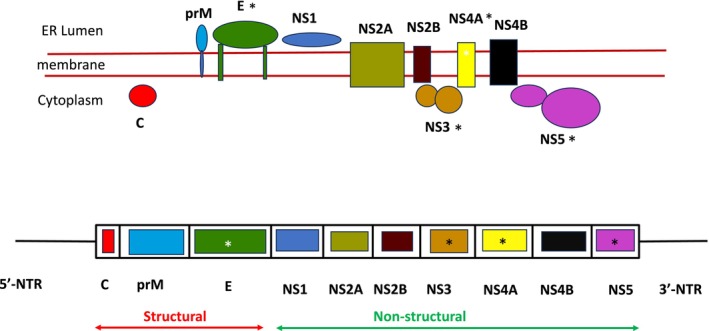
Genome map of West Nile Virus at the bottom, with the genome segments encoding the structural proteins C, M and E and the nonstructural proteins NS1, NS2A, NS2B, NS3, NS4A, NS4B and NS5. The genome is translated into a polyprotein and subsequently cleaved into the indicated individual proteins. The genome map and cleavage are simplified and not to scale. *Indicates mutations with phenotypic consequences discussed in the text. At the top is a scheme of the membrane topology for the West Nile Virus proteins as observed within the infected cell across the ER (endoplasmic reticulum) membrane. Figure credit: Simplified redrawing from Lindenbach et al. (2021).

Two hypotheses have been formulated to explain the more serious clinical symptoms of human WNV infection in North America compared to infections in the Old World: it could reflect an intrinsic higher virulence of the NY99 strain introduced into North America or its introduction into an immunologically naïve population. Data from experimental WNV infections in the American crow seem to favour the first hypothesis. A WNV isolate from Africa induced a delayed and 10,000‐fold lower viremia and a delayed and lower rate of mortality in American crows compared to crows infected with NY99 (31% vs. 94% death, respectively). Phylogenetic analysis of WNV genomes revealed a positive selection for aa position 249 in the nonstructural protein NS3 that correlated with higher virulence in crows. Specifically, NY99 showed a T249P replacement (Figure 3). When the reciprocal mutation P249T was introduced into NY99, its virulence in crows was attenuated. The NS3 T249P mutations have been introduced several times and independently in lineage I WNV strains; in two cases, it was associated with virulent human epidemics (Romania 1996 and Russia 1999, see below) (Brault et al. 2007).

## 2 WNV Infections in Europe

### 2.1 Major Outbreaks

Until 1962, WNV outbreaks were unknown in Europe. Then, France reported an epizootic in horses from the Camargue region. The affected animals showed neurological symptoms (ataxia and weakness) and a 25% mortality (Joubert et al. 1970). Several human cases of encephalitis and one death were also reported (Panthier et al. 1968). Serosurveys indicated that WNV continued to circulate in Europe without, however, causing clinical outbreaks. The situation changed in 1996 when a large epidemic was reported in Romania. Between July and October, 800 patients with neurological infections were counted in Bucharest and the Lower Danube valley. For 440 cases, diagnostic material was obtained and WNV infection was serologically confirmed in 80%. Meningitis (40%) and encephalitis (16%) were the dominant neurological signs. The disease started with abrupt fever, headache and neck stiffness. The fatality rate was 4%. Disease incidence and death risk increased substantially with age. The disease incidence was 12 per 100,000 people in Bucharest. WNV seroprevalence was not detected before the outbreak and increased to 4% in Bucharest after the outbreak, suggesting one clinical case for 140–320 subclinical infections. WNV was identified in *Cx. pipiens* mosquitoes. Houses, leaking pipes and poultry sheds were heavily infested with *Cx. pipiens*. WNV neutralising antibodies were detected in 40% of domestic fowl and in 8% of wild birds. The authors of the study suspected the introduction of WNV by migratory birds from the Middle East via the Black Sea flyway (Tsai et al. 1998). In 1998, a small WNV outbreak was reported among 14 horses from Tuscany/Italy, which developed ataxia and muscle weakness with recumbency; six horses died (Cantile et al. 2000). In 1999, a meningoencephalitis outbreak with 800 human cases was noted in Volgograd/Russia; 180 cases were serologically identified as WNV infections, and 40 of them were fatal. Virus sequences could be amplified by RT‐PCR from brain tissues of autopsied patients, demonstrating over the WNV E gene a sequence identical to that of the Romanian isolates (Platonov et al. 2001).

### 2.2 WNV Lineages

Before 2004, WNV infections in animals and humans from Europe displayed lineage 1 genome sequences, closely related to the North American WNV strains and WNV isolates from Africa, Asia (lineage 1a) and Australia (‘Kunjin virus’, lineage 1b). In the Czech Republic lineage 3 viruses circulated, but they were restricted to mosquitoes. In 2004, a lineage 2 WNV, normally circulating in sub‐Saharan Africa, was detected in Hungary, parallel to a local epizootic in geese and 14 WNV encephalitic cases in humans. The researchers suspected an introduction by migratory birds roaming the Hungarian wet plains and overwintering in Central Africa (Bakonyi et al. 2006). In 2010, an epidemic with 191 serologically diagnosed neuroinvasive human WNV cases was noted in northern Greece, causing 32 deaths, all in elderly patients. Mosquitoes yielded a lineage 2 virus closely related to the Hungarian isolate (Papa et al. 2011). Lineage 2 WNV subsequently shifted northward to Germany and The Netherlands.

### 2.3 Northward March of WNV

Detailed genome sequencing efforts from birds and mosquitoes differentiated two clusters in lineage 2 WNV that spread to the west (Cluster A) and to the south (Cluster B) of Europe with distinct but high dispersal velocities of 90–200 km per year. This speed suggests propagation by bird movements because these distances are too long to be explained by the dispersal of *Culex* mosquitoes. Cluster A WNV showed a northward spread to Germany and The Netherlands (Koch et al. 2024). Key attractors to the north were areas with high crop density, livestock cultivation, urbanisation and wetlands with high farming density, favouring *Cx. pipiens* breeding opportunities and increasing air temperature after 2003 (Lu et al. 2024). WNV cases occurred in 38 horses and 5 humans from East Germany. WNV sequencing in infected *Cx. pipiens* documented a single virus introduction event (Ziegler et al. 2020). Hibernating *Cx. pipiens* yielded WNV, demonstrating that the virus can overwinter in Germany. The low prevalence of WNV infections in mosquitoes suggests, however, that WNV is still a hypoendemic infection in Germany (Kampen et al. 2021). In 2020, marked by an unusual local heat wave, a cluster of nine human WNV cases was observed in Leipzig/Germany; six patients showed neurological disease and one patient died (Pietsch et al. 2020). In parallel, six WNV cases were reported from The Netherlands; two showed neurological disease (Vlaskamp et al. 2020).

### 2.4 WNV In Spain

WNV has been detected in Spain since 2004. A major outbreak occurred in 2020. It started in August in Sevilla and extended then to surrounding areas in Andalusia and Extremadura. Overall, 77 WNV human cases were reported, 94% presented with neurological disease and 8 died. In parallel, more than 100 outbreaks in horses were reported in the Guadalquivir river marshes from Andalusia. The human cases showed lineage 1 WNV, which was also detected in birds, horses and *Culex* vectors. *Cx. perexiguus* vector activity was dramatically increased in 2020 in rice‐growing areas where many aquatic birds concentrate (García San Miguel Rodríguez‐Alarcón et al. 2021). The outbreak was not produced by a new virus introduction but by a strain that circulated in the area at least since 2013 (Ruiz‐López et al. 2023). Longitudinal analyses of the incidence of WNV in common coot birds and horses in the 2020 and 2024 outbreak areas suggest that WNV incidence is higher after mild winters (Magallanes et al. 2023, 2024).

The number of infections decreased in 2021 and 2022. Mild winter conditions in 2023 extended vector virus detection until November. A resurgence with 158 WNV cases and 20 deaths was observed in 2024 in the West Andalusia and Extremadura regions (in Spanish: https://www.sanidad.gob.es/areas/alertasEmergenciasSanitarias/preparacionRespuesta/docs/20250131_ERR_Nilo_Occidental.pdf). These cases were linked with lineage 1 WNV, while lineage 2 WNV cases were reported in East Andalusia. In 2024, the European CDC (ECDC) reported 1,436 human cases for Europe The main infection foci were in the Mediterranean area (Italy > Greece > Spain), with 125 deaths for all of Europe. Seventy percent of the cases presented with neurological symptoms. In addition, more than 490 WNV outbreaks were reported in horses, mostly in Germany (https://www.ecdc.europa.eu/en/west‐nile‐fever/surveillance‐and‐disease‐data/disease‐data‐ecdc).

### 2.5 US–Europe WNV Outbreak Comparison

WNV outbreaks in Europe remained localised, while the WNV epidemic spread rapidly in a wave‐like fashion across North America. One possible reason explaining this difference is that birds and mammals from Eurasia were exposed to WNV in the past and therefore display a level of immunity not seen in birds and mammals from the Western hemisphere where animals were immunologically naïve towards WNV. In Europe, WNV is known from 1980 in Ukraine, and serological evidence from the 1970s demonstrated its presence in Albania. We therefore cannot anticipate an immunologically naïve population for WNV in Europe, which might explain why a continuous wave‐like spread over Europe was not observed, but isolated outbreaks, limited in time and geographical space. American robins play an important role in the USA in spreading WNV with their migration to the south, thus propagating the infection to further areas (Kilpatrick et al. 2006b). In contrast, the ecologically corresponding bird species in Europe, the Eurasian blackbird ( *Turdus merula* ), is a non‐migratory species resident in urban areas. The Eurasian blackbird is in Europe, a highly preferred host for *Cx. pipiens* (Rizzoli et al. 2015; Tiron et al. 2021). Correspondingly, blackbirds showed a seroprevalence of over 90% against flavivirus during recent WNV outbreaks in Spain (Figuerola et al. 2022). Interestingly, the relationship between biodiversity and WNV incidence is also different in North America and Europe. While counties in the US hosting a higher number of wild bird species had a reduced incidence of WNV in humans (Swaddle and Calos 2008), the opposite relationship has been reported in Europe, with higher WNV seroprevalence being reported in localities with a richer avian community (Ferraguti et al. 2021), suggesting that the capacity to amplify WNV may widely differ between avian communities on the two continents. Other researchers have noted genetic differences between *Culex* vectors in Europe and the US (Fonseca et al. 2004). An analysis of polymorphic microsatellite markers and multi‐locus genotype analysis identified two distinct branches of European *Culex* vectors. There is a European aboveground and a belowground branch of *Cx. pipiens*. The latter branch is represented by a northern European mosquito population living in underground railway tunnels of great cities that bite humans readily. The aboveground branch is represented by mosquitoes from Southern Europe, North Africa and the Middle East, which bite preferentially birds. A high presence of hybrids between both bioforms in the US could explain a high contact rate between mosquitoes feeding on both birds and humans. However, these hybrids are also frequent in southern Europe (Ancient origin of an urban underground mosquito | bioRxiv). A wide range of *Culex* species has been reportedly involved in the transmission of WNV in the US, with their relative importance shifting geographically (Rochlin et al. 2019). This contrasts with a more uniform *Culex* population in Europe, which might also explain the different WNV epidemiology pattern observed on the two continents.

## 3 Climate Change Effects

### 3.1 Temperature: Entomological Studies

Several biological factors determine the transmission of arboviruses. These are insect vector competence, vector population density, feeding behaviour and flight range, as well as population density and susceptibility of the vertebrate amplification hosts. Physical factors also matter. In the focus of current interest is ambient temperature to assess the potential effect of global warming on disease transmission (Watts et al. 2021a). For arbovirus infection, an important parameter is the extrinsic incubation period, the median time from imbibing an infectious blood meal until infected females transmit WNV. Dissemination of WNV within the body of *Cx. pipiens* after a blood meal occurred quicker and in a higher number of mosquitoes and reached higher titres when the ambient temperature increased from 18°C to 30°C (Dohm et al. 2002). Studies with *Cx. tarsalis* exposed to WNV NY99 demonstrated that donor birds need to exceed a WNV titre of 10^6^ pfu/mL blood to achieve a 50% infection rate of the vector. The mean titre in the vector increased as a function of incubation time in the vector and increased gradually with increasing temperatures up to 30°C. Only mosquitoes with WNV titres > 10^3^ pfu could transmit infections. Transmission efficiency increased with days after an infectious blood meal and increasing temperature. No transmission was detected below 18°C, and transmission was maximal at 26°C–30°C. WNV transmission and infection epicentres during the US 2002–2004 WNV epidemic were associated with regions showing above‐average summer temperatures (Reisen et al. 2006). Another study confirmed that the fraction of *Cx. pipiens* transmitting WNV was strongly temperature‐dependent: At 32°C, half of the mosquitoes transmitted WNV within 10 days after ingestion of an infectious blood meal; at 22°C, it took 4 weeks to reach this level of transmission. In addition, differences between WNV genotypes became apparent: WN02 transmitted 2‐fold more efficiently than NY99. At 18°C, only negligible transmission was observed (Kilpatrick et al. 2008). Shocket et al. (2020) analysed the temperature dependence of WNV transmission in three mosquito species (*Cx. pipiens* from temperate higher latitude areas, *Cx. quinquefasciatus* from temperate and tropical lower latitude areas, and *Cx. tarsalis* restricted to North and Central America covering temperate to tropical areas). Temperature had distinct effects on different biological traits: maximal mosquito development occurred at 30°C, bite rates peaked above 30°C, while life span decreased dramatically above 20°C. Oviposition was optimal at 25°C. WNV development rate within mosquitoes was maximal near 40°C, but thermal optima for transmission WNV was around 25°C (minimum 12°C–17°C and maximum 32°C–35°C according to different *Culex* species). The researchers concluded that climate warming will likely shift transmission of WNV diseases, increasing it in cooler locations while decreasing it in warmer locations.

### 3.2 Temperature: Epidemiological Studies and Predictions

Small local epidemiological investigations supported the importance of temperature for WNV activity. When analysing data from three counties in California, temperature and precipitation were the strongest predictors for the incidence of human WNV cases, followed by the proximity of WNV‐positive mosquito populations in neglected swimming pools (Hernandez et al. 2019). Water butts in urban gardens in the UK were dominantly colonised by *Cx. pipiens*. Titres were greater than in rural garden water butts, possibly explained by the urban heat island effect (Townroe and Callaghan 2014). While these data present anecdotal evidence for the importance of temperature on WNV infections, large data sets also concur with this conclusion. Epidemiologists analysed the 7‐fold higher WNV epidemic activity in Europe in 2018 compared to the preceding year. They used machine learning, combined with artificial intelligence trained on datasets from 2010 to 2017. In their analysis, key drivers for the 2018 peak epidemic activity were the mean temperature of the warmest quarter of the year, followed by maximum spring temperatures. Also, the preceding year's climate conditions made significant contributions (Farooq et al. 2022). These authors also tried predictions of WNV infections in Europe under climate change conditions for the time period 2040–2060. Under the most conservative conditions, the overall risk will increase by 3.5‐fold; Western Europe will see a marked 10‐fold increase. Lesser increases are foreseen for the Balkans and Southern Europe since in these regions already a third of the areas are already now at risk of infection. Risk will not increase linearly, but Europe will likely see sharp changes from year to year (Farooq et al. 2023). Although the authors also analysed the relationship between WNV incidence and vectors and host abundance, the poor quality of the original databases makes their results less reliable than for other climatic and environmental factors (Taheri et al. 2024). This criticism highlights the need to obtain reliable information on biodiversity at a European scale to improve our knowledge and allow projections for zoonotic pathogens. Based on data from 2010 and 2012, when major WNV outbreaks were noted in Volgograd, Russia and North Eastern Greece, high precipitation in late winter/early spring, high summer temperatures, summer drought, occurrence of irrigated croplands and highly fragmented forests were key predictors of WNV fever outbreaks (Marcantonio et al. 2015). Based on WNV outbreaks and spatial–temporal data for Europe from 2007 to 2018, Spanish researchers identified mean summer temperatures above 22°C, mean winter temperatures between 2°C and 6°C and rainy days in summer as meteorological factors, which are positively associated with WNV infections. These factors are known to further both the fitness and survival of *Culex* vectors (Watts et al. 2021b). Belgian researchers also analysed WNV data for the same time period for Europe and concurred with these observations. Air temperature in summer, followed by air temperature in winter, relative humidity in winter, precipitation in summer and relative humidity in fall affected the rate of WNV infections. They concluded that current hotspots of WNV circulation in Europe can largely be explained by climate change effects (Erazo et al. 2024).

When plotting the WNV cases between 2005 and 2019 on the US map, researchers observed a clustering along the population‐rich areas of the east and west coasts. However, when normalising for population size, a distinct pattern emerged. High WNV incidence areas described a wedge starting with a broad basis in the Great Plains at the Canadian border and a sharp end in Texas. A second hotspot was in Oregon/Idaho. Climate wise, these regions of high WNV incidence are characterised by dry (< 25 mm precipitation per month) and cold (mean below 0°C) winters. WNV incidence was low in areas with summer temperatures above 24°C and higher fall precipitation. Dry agricultural areas, supported by irrigation, bring birds, mosquitoes and humans into close proximity. *Cx. tarsalis* is a vector for WNV under drier conditions. American robins are present year‐round in the Great Plains. The Great Plains are a primary area for agriculture; people are therefore more likely outdoors for their occupation (Gorris et al. 2023).

The analysis of environmental factors affecting WNV outbreaks is complicated by the fact that infection does not only depend on *Culex* mosquitoes but also depend on bird biology. However, while 40% of the published reports investigated the infection‐related ecology of insect vectors, only 8% of the publications investigated birds, the major WNV reservoir. The authors concluded that more bird WNV ecology data are needed to achieve reliable predictions of climate and landscape effects on future WNV development in Europe and elsewhere (Gorris et al. 2023).

## 4 Outlook

A better understanding of WNV infections and their geographical spread needs data input from many different scientific disciplines, ranging from virology, over entomology to ornithology, veterinary and human medicine, epidemiology and climate science. Knowledge in this wide range of disciplines is rarely found represented in a single research department. Climate changes affect the biological cycle of viruses, the life traits and geographical distribution of mosquitoes and birds (serving as primary reservoirs), as well as behavioural responses of mammals and humans (serving as dead‐end hosts of medical importance). Climate is composed of many different physical factors, and the different biological systems will not respond in a parallel way to changing individual physical parameters, which will result in complex system responses with climate change, making the analysis and, even more, the prediction of future trends a challenging task. Landscape structure affected by urbanisation and agricultural activities also prominently influences the ecology of insects and birds, adding an additional layer of complexity to prediction models. WNV infections are thus a typical task for the One Health Initiative (Giesen et al. 2023), coordinated by four United Nations (UN) organisations: FAO (Food and Agricultural Organisation), UNEP (UN Environment Program), WHO (World Health Organisation) and WOAH (World Organisation for Animal Health), treating human, animal and environmental health as one integral objective (One Health). In view of the limited financial resources of these UN organisations, One Health initiatives also need the support of the international scientific community conducting research work in the field of One Health questions. As progress in this field depends on data acquisition from time‐intensive field work, the help of citizen scientists is here absolutely needed for collecting data on mosquitoes and birds (Ainsworth 2023). Citizen scientists could also spread the information on climate effects on disease spread to a wider public, which will be important to increase the acceptance for future control measures and motivation for changes in individual behaviour to curtail the likely increased risk of arbovirus infections in the future.

The Lancet Countdown on health and climate change concluded that climate change is affecting the distribution and risk of many infectious diseases to humans, particularly vector‐, food‐ and water‐borne diseases. The report expressively mentioned infections with dengue virus, falciparum malaria parasites and *Vibrio* bacteria, noting that climate suitability for disease transmission is increasing globally (Watts et al. 2021a). A New England Journal of Medicine review on climate change and vector‐borne diseases added Lyme disease and WNV disease to this list (Thomson and Stanberry 2022). Since WNV disease affects populations in the industrialised Northern hemisphere while vaccines and curative treatments are currently lacking, the political and financial incentives are given to increase academic and industrial research on mosquito‐vectored infectious disease.

## Author Contributions

**Harald Brüssow:** conceptualization, writing – original draft, visualization, investigation. **Jordi Figuerola:** validation, formal analysis, investigation.

## Conflicts of Interest

The authors declare no conflicts of interest.

## Data Availability

Data sharing not applicable to this article as no datasets were generated or analysed during the current study.

## References

[mbt270120-bib-0001] Aguilera‐Sepúlveda, P. , C. Cano‐Gómez , R. Villalba , et al. 2024. “The Key Role of Spain in the Traffic of West Nile Virus Lineage 1 Strains Between Europe and Africa.” Infectious Disease 56, no. 9: 743–758. 10.1080/23744235.2024.2348633. 38836293

[mbt270120-bib-0002] Ainsworth, C. 2023. “Tropical Diseases Move North.” Nature. 10.1038/d41586-023-03476-7. 37945701

[mbt270120-bib-0003] Anderson, J. F. , T. G. Andreadis , C. R. Vossbrinck , et al. 1999. “Isolation of West Nile Virus From Mosquitoes, Crows, and a Cooper's Hawk in Connecticut.” Science 286, no. 5448: 2331–2333. 10.1126/science.286.5448.2331. 10600741

[mbt270120-bib-0004] Bakonyi, T. , E. Ivanics , K. Erdélyi , et al. 2006. “Lineage 1 and 2 Strains of Encephalitic West Nile Virus, Central Europe.” Emerging Infectious Diseases 12, no. 4: 618–623. 10.3201/eid1204.051379. 16704810 PMC3294705

[mbt270120-bib-0005] Baty, S. A. , K. B. Gibney , J. E. Staples , et al. 2012. “Evaluation for West Nile Virus (WNV) RNA in Urine of Patients Within 5 Months of WNV Infection.” Journal of Infectious Diseases 205, no. 9: 1476–1477. 10.1093/infdis/jis221. 22438324

[mbt270120-bib-0006] Brault, A. C. , C. Y. Huang , S. A. Langevin , et al. 2007. “A Single Positively Selected West Nile Viral Mutation Confers Increased Virogenesis in American Crows.” Nature Genetics 39, no. 9: 1162–1166. 10.1038/ng2097. 17694056 PMC2291521

[mbt270120-bib-0007] Busch, M. P. , S. Caglioti , E. F. Robertson , et al. 2005. “Screening the Blood Supply for West Nile Virus RNA by Nucleic Acid Amplification Testing.” New England Journal of Medicine 353, no. 5: 460–467. 10.1056/NEJMoa044029. 16079369

[mbt270120-bib-0008] Cantile, C. , G. Di Guardo , C. Eleni , and M. Arispici . 2000. “Clinical and Neuropathological Features of West Nile Virus Equine Encephalomyelitis in Italy.” Equine Veterinary Journal 32, no. 1: 31–35. 10661382 10.2746/042516400777612080

[mbt270120-bib-0009] Davis, C. T. , G. D. Ebel , R. S. Lanciotti , et al. 2005. “Phylogenetic Analysis of North American West Nile Virus Isolates, 2001‐2004: Evidence for the Emergence of a Dominant Genotype.” Virology 342, no. 2: 252–265. 10.1016/j.virol.2005.07.022. 16137736

[mbt270120-bib-0010] Deardorff, E. R. , K. A. Fitzpatrick , G. V. Jerzak , P. Y. Shi , L. D. Kramer , and G. D. Ebel . 2011. “West Nile Virus Experimental Evolution In Vivo and the Trade‐Off Hypothesis.” PLoS Pathogens 7, no. 11: e1002335. 10.1371/journal.ppat.1002335. 22102808 PMC3213084

[mbt270120-bib-0011] Dohm, D. J. , M. L. O'Guinn , and M. J. Turell . 2002. “Effect of Environmental Temperature on the Ability of *Culex pipiens* (Diptera: Culicidae) to Transmit West Nile Virus.” Journal of Medical Entomology 39, no. 1: 221–225. 10.1603/0022-2585-39.1.221. 11931261

[mbt270120-bib-0012] Duggal, N. K. , A. Bosco‐Lauth , R. A. Bowen , et al. 2014. “Evidence for Co‐Evolution of West Nile Virus and House Sparrows in North America.” PLoS Neglected Tropical Diseases 8, no. 10: e3262. 10.1371/journal.pntd.0003262. 25357248 PMC4214623

[mbt270120-bib-0013] Erazo, D. , L. Grant , G. Ghisbain , et al. 2024. “Contribution of Climate Change to the Spatial Expansion of West Nile Virus in Europe.” Nature Communications 15, no. 1: 1196. 10.1038/s41467-024-45290-3. PMC10853512 38331945

[mbt270120-bib-0014] Farooq, Z. , J. Rocklöv , J. Wallin , et al. 2022. “Artificial Intelligence to Predict West Nile Virus Outbreaks With Eco‐Climatic Drivers.” Lancet Regional Health 17: 100370. 10.1016/j.lanepe.2022.100370. PMC8971633 35373173

[mbt270120-bib-0015] Farooq, Z. , H. Sjödin , J. C. Semenza , et al. 2023. “European Projections of West Nile Virus Transmission Under Climate Change Scenarios.” One Health 16: 100509. 10.1016/j.onehlt.2023.100509. 37363233 PMC10288058

[mbt270120-bib-0016] Ferraguti, M. , J. Martínez‐de la Puente , M. Á. Jiménez‐Clavero , et al. 2021. “A Field Test of the Dilution Effect Hypothesis in Four Avian Multi‐Host Pathogens.” PLoS Pathogens 17, no. 6: e1009637. 10.1371/journal.ppat.1009637. 34161394 PMC8221496

[mbt270120-bib-0017] Figuerola, J. , M. Á. Jiménez‐Clavero , M. J. Ruíz‐López , et al. 2022. “A One Health View of the West Nile Virus Outbreak in Andalusia (Spain) in 2020.” Emerging Microbes & Infections 11, no. 1: 2570–2578. 10.1080/22221751.2022.2134055. 36214518 PMC9621199

[mbt270120-bib-0018] Fonseca, D. M. , N. Keyghobadi , C. A. Malcolm , et al. 2004. “Emerging Vectors in the *Culex pipiens* Complex.” Science 303, no. 5663: 1535–1538. 10.1126/science.1094247. 15001783

[mbt270120-bib-0019] García San Miguel Rodríguez‐Alarcón, L. , B. Fernández‐Martínez , M. J. Sierra Moros , et al. 2021. “Unprecedented Increase of West Nile Virus Neuroinvasive Disease, Spain, Summer 2020.” Euro Surveillance 26, no. 19: 2002010. 10.2807/1560-7917. 33988123 PMC8120797

[mbt270120-bib-0020] George, T. L. , R. J. Harrigan , J. A. LaManna , D. F. DeSante , J. F. Saracco , and T. B. Smith . 2015. “Persistent Impacts of West Nile Virus on North American Bird Populations.” Proceedings of the National Academy of Sciences of the United States of America 112, no. 46: 14290–14294. 10.1073/pnas.1507747112. 26578774 PMC4655513

[mbt270120-bib-0021] Giesen, C. , Z. Herrador , B. Fernandez‐Martinez , et al. 2023. “A Systematic Review of Environmental Factors Related to WNV Circulation in European and Mediterranean Countries.” One Health 16: 100478. 10.1016/j.onehlt.2022.100478. 37363246 PMC10288031

[mbt270120-bib-0022] Goldblum, N. , W. Jasinska‐Klingberg , M. A. Klingberg , K. Marberg , and V. V. Sterk . 1956. “The Natural History of West Nile Fever. I. Clinical Observations During an Epidemic in Israel.” American Journal of Hygiene 64, no. 3: 259–269. 10.1093/oxfordjournals.aje.a119838. 13372519

[mbt270120-bib-0023] Gorris, M. E. , J. T. Randerson , S. R. Coffield , et al. 2023. “Assessing the Influence of Climate on the Spatial Pattern of West Nile Virus Incidence in the United States.” Environmental Health Perspectives 131, no. 4: 47016. 10.1289/EHP10986. 37104243 PMC10137712

[mbt270120-bib-0024] Hadfield, J. , A. F. Brito , D. M. Swetnam , et al. 2019. “Twenty Years of West Nile Virus Spread and Evolution in the Americas Visualized by Nextstrain.” PLoS Pathogens 15, no. 10: e1008042. 10.1371/journal.ppat.1008042. 31671157 PMC6822705

[mbt270120-bib-0025] Hamel, R. , Q. Narpon , I. Serrato‐Pomar , et al. 2024. “West Nile Virus Can Be Transmitted Within Mosquito Populations Through Infectious Mosquito Excreta.” iScience 27, no. 11: 111099. 10.1016/j.isci.2024.111099. 39473977 PMC11513527

[mbt270120-bib-0026] Hernandez, E. , R. Torres , and A. L. Joyce . 2019. “Environmental and Sociological Factors Associated With the Incidence of West Nile Virus Cases in the Northern San Joaquin Valley of California, 2011‐2015.” Vector Borne and Zoonotic Diseases 19, no. 11: 851–858. 10.1089/vbz.2019.2437. 31211639 PMC6818473

[mbt270120-bib-0078] Higgs, S. , B. S. Schneider , D. L. Vanlandingham , K. A. Klingler , and E. A. Gould . 2005. “Nonviremic transmission of West Nile virus.” Proceedings of the National Academy of Sciences of the United States of America 102, no. 25: 8871–8874. 10.1073/pnas.0503835102. 15951417 PMC1157059

[mbt270120-bib-0027] Iwamoto, M. , D. B. Jernigan , A. Guasch , et al. 2003. “Transmission of West Nile Virus From an Organ Donor to Four Transplant Recipients.” New England Journal of Medicine 348, no. 22: 2196–2203. 10.1056/NEJMoa022987. 12773646

[mbt270120-bib-0028] Joubert, L. , J. Oudar , C. Hannoun , et al. 1970. “Epidemiologie du virus West Nile: etude d'un foyer en Camargue. IV. La meningo‐encephalomyelite du cheval.” Annales de l'Institut Pasteur 118, no. 2: 239–247. 5461277

[mbt270120-bib-0029] Kampen, H. , B. A. Tews , and D. Werner . 2021. “First Evidence of West Nile Virus Overwintering in Mosquitoes in Germany.” Viruses 13, no. 12: 2463. 10.3390/v13122463. 34960732 PMC8703620

[mbt270120-bib-0030] Kilpatrick, A. M. , P. Daszak , M. J. Jones , P. P. Marra , and L. D. Kramer . 2006a. “Host Heterogeneity Dominates West Nile Virus Transmission.” Proceedings of the Biological Sciences 273, no. 1599: 2327–2333. 10.1098/rspb.2006.3575. PMC1636093 16928635

[mbt270120-bib-0031] Kilpatrick, A. M. , L. D. Kramer , S. R. Campbell , E. O. Alleyne , A. P. Dobson , and P. Daszak . 2005. “West Nile Virus Risk Assessment and the Bridge Vector Paradigm.” Emerging Infectious Diseases 11, no. 3: 425–429. 10.3201/eid1103.040364. 15757558 PMC3298247

[mbt270120-bib-0032] Kilpatrick, A. M. , L. D. Kramer , M. J. Jones , P. P. Marra , and P. Daszak . 2006b. “West Nile Virus Epidemics in North America Are Driven by Shifts in Mosquito Feeding Behavior.” PLoS Biology 4, no. 4: e82. 10.1371/journal.pbio.0040082. 16494532 PMC1382011

[mbt270120-bib-0033] Kilpatrick, A. M. , M. A. Meola , R. M. Moudy , and L. D. Kramer . 2008. “Temperature, Viral Genetics, and the Transmission of West Nile Virus by *Culex pipiens* Mosquitoes.” PLoS Pathogens 4, no. 6: e1000092. 10.1371/journal.ppat.1000092. 18584026 PMC2430533

[mbt270120-bib-0034] Kilpatrick, A. M. , and S. S. Wheeler . 2019. “Impact of West Nile Virus on Bird Populations: Limited Lasting Effects, Evidence for Recovery, and Gaps in Our Understanding of Impacts on Ecosystems.” Journal of Medical Entomology 56, no. 6: 1491–1497. 10.1093/jme/tjz149. 31549723 PMC6821264

[mbt270120-bib-0035] Koch, R. T. , D. Erazo , A. J. Folly , et al. 2024. “Genomic Epidemiology of West Nile Virus in Europe.” One Health 18: 100664. 10.1016/j.onehlt.2023.100664. 38193029 PMC10772404

[mbt270120-bib-0036] Komar, N. , and G. G. Clark . 2006. “West Nile Virus Activity in Latin America and the Caribbean.” Revista Panamericana de Salud Pública 19, no. 2: 112–117. 10.1590/s1020-49892006000200006. 16551385

[mbt270120-bib-0037] Komar, N. , S. Langevin , S. Hinten , et al. 2003. “Experimental Infection of North American Birds With the New York 1999 Strain of West Nile Virus.” Emerging Infectious Diseases 9, no. 3: 311–322. 10.3201/eid0903.020628. 12643825 PMC2958552

[mbt270120-bib-0038] LaDeau, S. L. , A. M. Kilpatrick , and P. P. Marra . 2007. “West Nile Virus Emergence and Large‐Scale Declines of North American Bird Populations.” Nature 447, no. 7145: 710–713. 10.1038/nature05829. 17507930

[mbt270120-bib-0039] Lanciotti, R. S. , J. T. Roehrig , V. Deubel , et al. 1999. “Origin of the West Nile Virus Responsible for an Outbreak of Encephalitis in the Northeastern United States.” Science 286, no. 5448: 2333–2337. 10.1126/science.286.5448.2333. 10600742

[mbt270120-bib-0040] Lindenbach, B. D. , G. Randall , R. Bartenschläger , and C. M. Rice . 2021. “Chapter 7: Flaviviridae: The Viruses and their Replication.” In Fields Virology: Emerging Viruses, edited by P. W. Howley and D. M. Knipe , vol. 1, 246–301. Wolters Kluwer.

[mbt270120-bib-0041] Lu, L. , F. Zhang , B. B. Oude Munnink , et al. 2024. “West Nile Virus Spread in Europe: Phylogeographic Pattern Analysis and Key Drivers.” PLoS Pathogens 20, no. 1: e1011880. 10.1371/journal.ppat.1011880. 38271294 PMC10810478

[mbt270120-bib-0042] Magallanes, S. , F. Llorente , M. J. Ruiz‐López , et al. 2023. “Long‐Term Serological Surveillance for West Nile and Usutu Virus in Horses in South‐West Spain.” One Health 17: 100578. 10.1016/j.onehlt.2023.100578. 38024263 PMC10665154

[mbt270120-bib-0043] Magallanes, S. , F. Llorente , M. J. Ruiz‐López , et al. 2024. “Warm Winters Are Associated to More Intense West Nile Virus Circulation in Southern Spain.” Emerging Microbes & Infections 13, no. 1: 2348510. 10.1080/22221751.2024.2348510. 38686545 PMC11073421

[mbt270120-bib-0044] Marcantonio, M. , A. Rizzoli , M. Metz , et al. 2015. “Identifying the Environmental Conditions Favouring West Nile Virus Outbreaks in Europe.” PLoS One 10, no. 3: e0121158. 10.1371/journal.pone.0121158. 25803814 PMC4372576

[mbt270120-bib-0045] Marfin, A. A. , L. R. Petersen , M. Eidson , et al. 2001. “Widespread West Nile Virus Activity, Eastern United States, 2000.” Emerging Infectious Diseases 7, no. 4: 730–735. 10.3201/eid0704.010423. 11585539 PMC2631748

[mbt270120-bib-0046] Molaei, G. , T. G. Andreadis , P. M. Armstrong , J. F. Anderson , and C. R. Vossbrinck . 2006. “Host Feeding Patterns of Culex Mosquitoes and West Nile Virus Transmission, Northeastern United States.” Emerging Infectious Diseases 12, no. 3: 468–474. 10.3201/eid1203.051004. 16704786 PMC3291451

[mbt270120-bib-0047] Morales, M. A. , M. Barrandeguy , C. Fabbri , et al. 2006. “West Nile Virus Isolation From Equines in Argentina, 2006.” Emerging Infectious Diseases 12, no. 10: 1559–1561. 10.3201/eid1210.060852. 17176571 PMC3290965

[mbt270120-bib-0048] Mostashari, F. , M. L. Bunning , P. T. Kitsutani , et al. 2001. “Epidemic West Nile Encephalitis, New York, 1999: Results of a Household‐Based Seroepidemiological Survey.” Lancet 358, no. 9278: 261–264. 10.1016/S0140-6736(01)05480-0. 11498211

[mbt270120-bib-0049] Moudy, R. M. , M. A. Meola , L. L. Morin , G. D. Ebel , and L. D. Kramer . 2007. “A Newly Emergent Genotype of West Nile Virus Is Transmitted Earlier and More Efficiently by Culex Mosquitoes.” American Journal of Tropical Medicine and Hygiene 77, no. 2: 365–370. 17690414

[mbt270120-bib-0050] Murray, K. , C. Walker , E. Herrington , et al. 2010. “Persistent Infection With West Nile Virus Years After Initial Infection.” Journal of Infectious Diseases 201, no. 1: 2–4. 10.1086/648731. 19961306 PMC2791189

[mbt270120-bib-0051] Murray, K. O. , D. Ruktanonchai , D. Hesalroad , E. Fonken , and M. S. Nolan . 2013. “West Nile Virus, Texas, USA, 2012.” Emerging Infectious Diseases 19, no. 11: 1836–1838. 10.3201/eid1911.130768. 24210089 PMC3837649

[mbt270120-bib-0052] Nasci, R. S. , D. J. White , H. Stirling , et al. 2001. “West Nile Virus Isolates From Mosquitoes in New York and New Jersey, 1999.” Emerging Infectious Diseases 7, no. 4: 626–630. 10.3201/eid0704.010404. 11585523 PMC2631761

[mbt270120-bib-0053] Nash, D. , F. Mostashari , A. Fine , et al. 2001. “The Outbreak of West Nile Virus Infection in the new York City Area in 1999.” New England Journal of Medicine 344, no. 24: 1807–1814. 10.1056/NEJM200106143442401. 11407341

[mbt270120-bib-0054] Ndiva Mongoh, M. , R. Hearne , N. W. Dyer , and M. L. Khaitsa . 2008. “The Economic Impact of West Nile Virus Infection in Horses in the North Dakota Equine Industry in 2002.” Tropical Animal Health and Production 40, no. 1: 69–76. 10.1007/s11250-007-9055-8. 18551781

[mbt270120-bib-0055] Panthier, R. , C. Hannoun , D. Beytout , and J. Mouchet . 1968. “Epidemiologie du virus west nile. Etudes d'un foyer en Camargue. 3.‐Les maladies humaines.” Annales de l'lnstitut Pasteur 115, no. 3: 435–445. 5711530

[mbt270120-bib-0056] Papa, A. , T. Bakonyi , K. Xanthopoulou , A. Vázquez , A. Tenorio , and N. Nowotny . 2011. “Genetic Characterization of West Nile Virus Lineage 2, Greece, 2010.” Emerging Infectious Diseases 17, no. 5: 920–922. 10.3201/eid1705.101759. 21529413 PMC3321789

[mbt270120-bib-0057] Pealer, L. N. , A. A. Marfin , L. R. Petersen , et al. 2003. “Transmission of West Nile Virus Through Blood Transfusion in the United States in 2002.” New England Journal of Medicine 349, no. 13: 1236–1245. 10.1056/NEJMoa030969. 14500806

[mbt270120-bib-0058] Pearson, T. C. , H. M. Lazear , and M. S. Diamond . 2021. “Flaviviruses: Dengue, Zika, West Nile, Yellow Fever and Other Flaviviruses.” In Fields Virology Vol. 1: Emerging Viruses, edited by P. M. Howley , D. M. Knipe , and S. P. Whelan , 345–409. Wolters Kluwer Publisher.

[mbt270120-bib-0059] Petersen, L. R. , P. J. Carson , B. J. Biggerstaff , B. Custer , S. M. Borchardt , and M. P. Busch . 2013. “Estimated Cumulative Incidence of West Nile Virus Infection in US Adults, 1999‐2010.” Epidemiology and Infection 141, no. 3: 591–595. 10.1017/S0950268812001070. 22640592 PMC9151873

[mbt270120-bib-0079] Pietsch, C. , D. Michalski , J. Münch , et al. 2020. “Autochthonous West Nile virus infection outbreak in humans, Leipzig, Germany, August to September 2020.” Eurosurveillance 25, no. 46: 2001786. 10.2807/1560-7917.ES.2020.25.46.2001786. 33213686 PMC7678033

[mbt270120-bib-0060] Platonov, A. E. , G. A. Shipulin , O. Y. Shipulina , et al. 2001. “Outbreak of West Nile Virus Infection, Volgograd Region, Russia, 1999.” Emerging Infectious Diseases 7, no. 1: 128–132. 10.3201/eid0701.010118. 11266303 PMC2631674

[mbt270120-bib-0061] Reisen, W. K. , Y. Fang , and V. M. Martinez . 2006. “Effects of Temperature on the Transmission of West Nile Virus by *Culex Tarsalis* (Diptera: Culicidae).” Journal of Medical Entomology 43, no. 2: 309–317. 10.1603/0022-2585(2006)043[0309:EOTOTT]2.0.CO;2. 16619616

[mbt270120-bib-0062] Rizzoli, A. , L. Bolzoni , E. A. Chadwick , et al. 2015. “Understanding West Nile Virus Ecology in Europe: *Culex pipiens* Host Feeding Preference in a Hotspot of Virus Emergence.” Parasites & Vectors 8: 213. 10.1186/s13071-015-0831-4. 25888754 PMC4411713

[mbt270120-bib-0063] Rochlin, I. , A. Faraji , K. Healy , and T. G. Andreadis . 2019. “West Nile Virus Mosquito Vectors in North America.” Journal of Medical Entomology 56, no. 6: 1475–1490. 10.1093/jme/tjz146. 31549725

[mbt270120-bib-0064] Ronca, S. E. , J. C. Ruff , and K. O. Murray . 2021. “A 20‐Year Historical Review of West Nile Virus Since Its Initial Emergence in North America: Has West Nile Virus Become a Neglected Tropical Disease?” PLoS Neglected Tropical Diseases 15, no. 5: e0009190. 10.1371/journal.pntd.0009190. 33956816 PMC8101735

[mbt270120-bib-0065] Ruiz‐López, M. J. , M. Muñoz‐Chimeno , J. Figuerola , et al. 2023. “Genomic Analysis of West Nile Virus Lineage 1 Detected in Mosquitoes During the 2020‐2021 Outbreaks in Andalusia, Spain.” Viruses 15, no. 2: 266. 10.3390/v15020266. 36851481 PMC9962355

[mbt270120-bib-0066] Shocket, M. S. , A. B. Verwillow , M. G. Numazu , et al. 2020. “Transmission of West Nile and Five Other Temperate Mosquito‐Borne Viruses Peaks at Temperatures Between 23°C and 26°C.” eLife 9: e58511. 10.7554/eLife.58511. 32930091 PMC7492091

[mbt270120-bib-0067] Stramer, S. L. , C. T. Fang , G. A. Foster , A. G. Wagner , J. P. Brodsky , and R. Y. Dodd . 2005. “West Nile Virus Among Blood Donors in the United States, 2003 and 2004.” New England Journal of Medicine 353, no. 5: 451–459. 10.1056/NEJMoa044333. 16079368

[mbt270120-bib-0068] Swaddle, J. P. , and S. E. Calos . 2008. “Increased Avian Diversity Is Associated With Lower Incidence of Human West Nile Infection: Observation of the Dilution Effect.” PLoS One 3, no. 6: e2488. 10.1371/journal.pone.0002488. 18575599 PMC2427181

[mbt270120-bib-0069] Taheri, S. , M. J. Ruiz‐López , S. Magallanes , and J. Figuerola . 2024. “Input Precision, Output Excellence: The Importance of Data Quality Control and Method Selection in Disease Risk Mapping.” Lancet Regional Health Europe 42: 100944. 10.1016/j.lanepe.2024.100944. 38831798 PMC11144752

[mbt270120-bib-0070] Thomson, M. C. , and L. R. Stanberry . 2022. “Climate Change and Vectorborne Diseases.” New England Journal of Medicine 387, no. 21: 1969–1978. 10.1056/NEJMra2200092. 36416768

[mbt270120-bib-0071] Tiron, G. V. , I. G. Stancu , S. Dinu , et al. 2021. “Characterization and Host‐Feeding Patterns of *Culex pipiens* s.l. Taxa in a West Nile Virus‐Endemic Area in Southeastern Romania.” Vector Borne and Zoonotic Diseases 21, no. 9: 713–719. 10.1089/vbz.2020.2739. 34160283

[mbt270120-bib-0072] Townroe, S. , and A. Callaghan . 2014. “British Container Breeding Mosquitoes: The Impact of Urbanisation and Climate Change on Community Composition and Phenology.” PLoS One 9, no. 4: e95325. 10.1371/journal.pone.0095325. 24759617 PMC3997353

[mbt270120-bib-0073] Tsai, T. F. , F. Popovici , C. Cernescu , G. L. Campbell , and N. I. Nedelcu . 1998. “West Nile Encephalitis Epidemic in Southeastern Romania.” Lancet 352, no. 9130: 767–771. 10.1016/s0140-6736(98)03538-7. 9737281

[mbt270120-bib-0074] Vlaskamp, D. R. , S. F. Thijsen , J. Reimerink , et al. 2020. “First Autochthonous Human West Nile Virus Infections in The Netherlands, July to August 2020.” Euro Surveillance 25, no. 46: 2001904. 10.2807/1560-7917.ES.2020.25.46.2001904. 33213687 PMC7678035

[mbt270120-bib-0075] Watts, M. J. , V. Sarto i Monteys , P. G. Mortyn , and P. Kotsila . 2021b. “The Rise of West Nile Virus in Southern and Southeastern Europe: A Spatial‐Temporal Analysis Investigating the Combined Effects of Climate, Land Use and Economic Changes.” One Health 13: 100315. 10.1016/j.onehlt.2021.100315. 34485672 PMC8408625

[mbt270120-bib-0076] Watts, N. , M. Amann , N. Arnell , et al. 2021a. “The 2020 Report of the Lancet Countdown on Health and Climate Change: Responding to Converging Crises.” Lancet 397: 129–170. 10.1016/S0140-6736(20)32290-X. 33278353 PMC7616803

[mbt270120-bib-0077] Ziegler, U. , P. D. Santos , M. H. Groschup , et al. 2020. “West Nile Virus Epidemic in Germany Triggered by Epizootic Emergence, 2019.” Viruses 12, no. 4: 448. 10.3390/v12040448. 32326472 PMC7232143

